# Supplementary figures and images for: Structural insights into SETD3-mediated histidine methylation on β-actin
Source: eLife. 2019 Feb 20;8:e43676. doi: 10.7554/eLife.43676 (PMC6400499; doi:10.7554/eLife.43676)

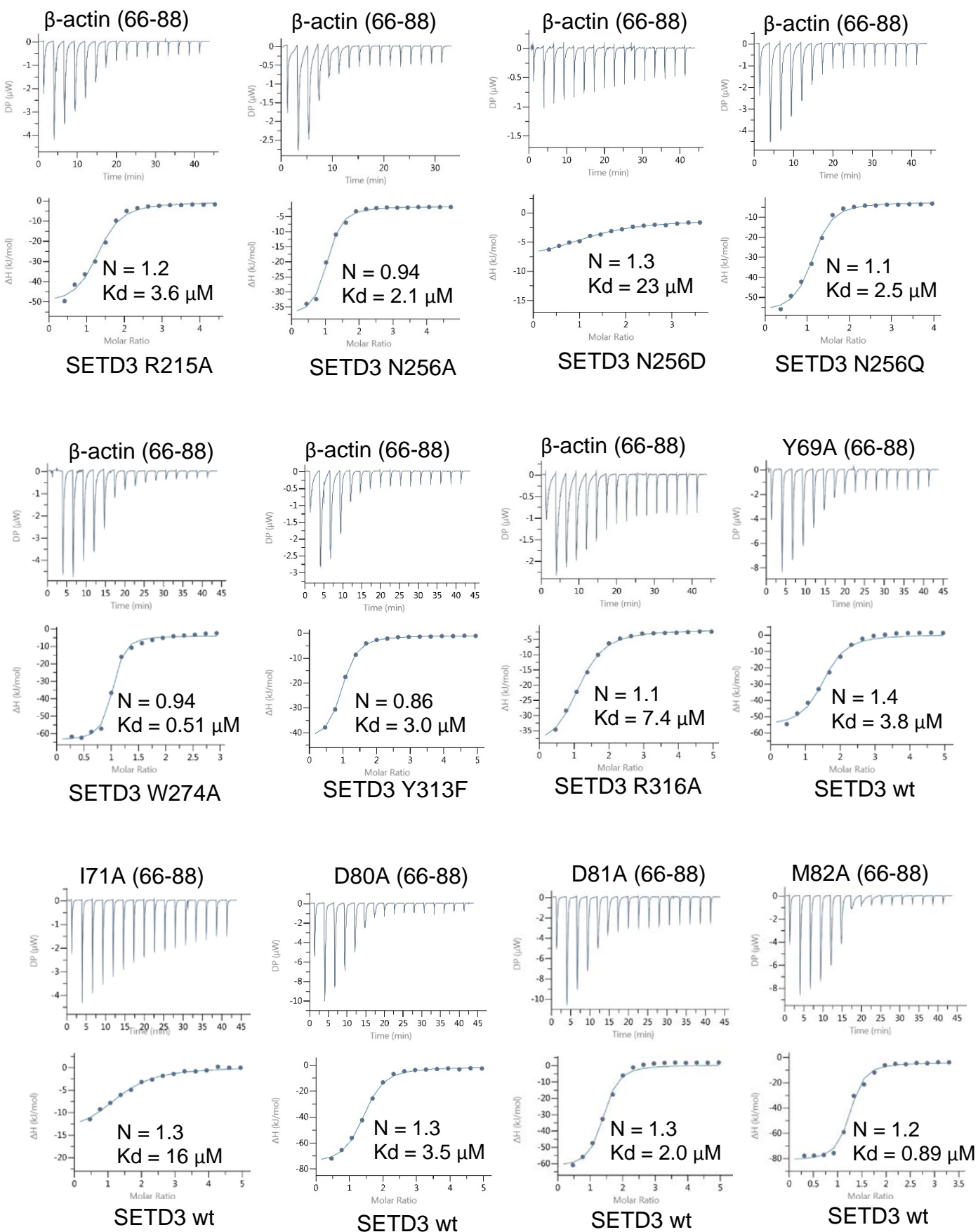

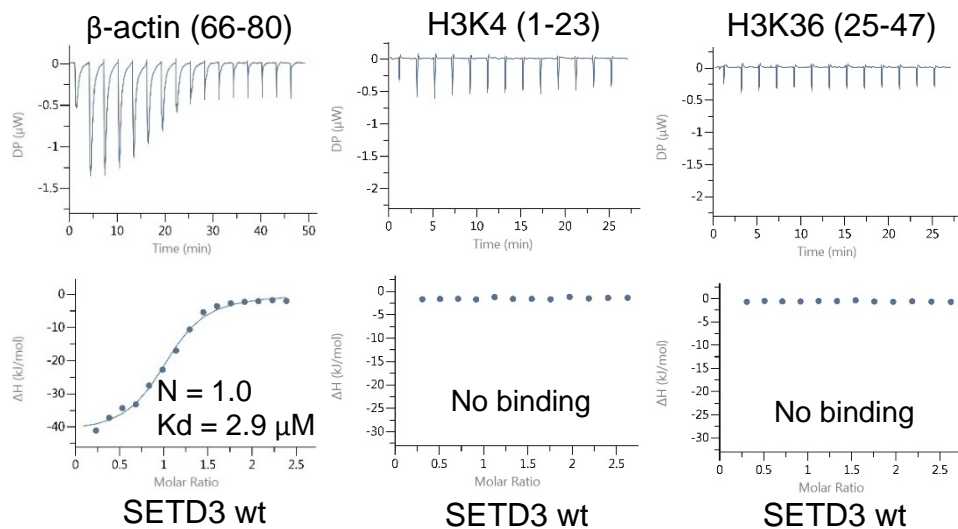

**Supplemental File 1.** ITC binding curves for the binding measurements reported in Table 1.

Supplement: Supplementary file 1. [file elife-43676-supp1.pdf]
